# HRD‐Driven Reprogramming of Macrophage Function and Spatial Architecture in High‐Grade Serous Ovarian Cancer

**DOI:** 10.1155/genr/7364793

**Published:** 2026-05-09

**Authors:** Hua Lan, Fang Xu, Linshuang Li, Xin Wei, Minghua Li

**Affiliations:** ^1^ Department of Gynecology and Obstetrics, Changsha Central Hospital of University of South China, Changsha, Hunan, China

**Keywords:** high-grade serous ovarian cancer (HGSOC), homologous recombination deficiency (HRD), macrophage reprogramming, tumor immune microenvironment

## Abstract

High‐grade serous ovarian cancer (HGSOC) exhibits homologous recombination deficiency (HRD), but its impact on the immune microenvironment remains unclear. Using single‐cell RNA sequencing, spatial transcriptomic inference, and survival analyses, we characterized 166,895 macrophages across HRD subtypes: functional BRCA1/2 inactivation (FBI), HRD‐Del (deletions), and HRD‐Dup (duplications). FBI macrophages showed lipid metabolism enrichment (S100A8, CD36), HRD‐Del macrophages upregulated antigen presentation (HLA‐DQA1, HLA‐DPB1), and HRD‐Dup macrophages displayed interferon‐stimulated gene expression (ISG15, MX1). Six macrophage subtypes (C1Q, FCN1, MARCO, MKI67, MMP9, S100A9) exhibited distinct spatial distributions and functions. MKI67+ macrophages correlated with improved survival, while C1Q + subsets predicted worse outcomes. HRD‐Dup tumors with high macrophage signatures had better survival, suggesting a favorable immune landscape. Our findings reveal HRD‐driven macrophage reprogramming as a key determinant of immune microenvironment composition and clinical outcomes, supporting HRD‐specific macrophage‐targeted therapies for HGSOC.

## 1. Introduction

High‐grade serous ovarian cancer (HGSOC) remains one of the most aggressive and deadly gynecological malignancies, largely due to its heterogeneity and immune evasion mechanisms [1]. Recent advances in single‐cell transcriptomics have facilitated an unprecedented understanding of the immune microenvironment dynamics, particularly the functional diversity of macrophages, which play critical roles in tumor progression, immune modulation, and therapeutic resistance [2]. The key genomic drivers of HGSOC are alterations in homologous recombination repair, which are classified into functional BRCA1/2 inactivation (FBI), homologous recombination deficiency (HRD) due to deletions (HRD‐Del), and HRD due to duplications (HRD‐Dup) [3, 4]. Although HRD‐driven genomic instability is well‐established as a critical factor influencing tumor biology and therapeutic response, its impact on macrophage phenotypes and spatial architecture within the tumor immune microenvironment remains poorly understood [5].

Macrophages exhibit remarkable plasticity and functional specialization within the tumor microenvironment [6, 7]. From antigen presentation and immune activation to extracellular matrix remodeling and immunosuppression, macrophage subtypes are influenced by intrinsic tumor genomics, tissue‐specific cues, and spatial organization [8]. Nonetheless, the links between HRD‐driven genomic subtypes and macrophage functional diversity, spatial heterogeneity, and prognostic significance have not been comprehensively defined [9]. Bridging this gap holds immense potential for the identification of new macrophage‐based biomarkers and therapeutic strategies tailored to HRD‐specific immune microenvironment.

The current study aimed to elucidate the impact of HRD‐driven genomic alterations on macrophage function, heterogeneity, and localization in HGSOC using public single‐cell RNA sequencing (scRNA‐seq) data and spatial analyses. By classifying macrophage populations based on HRD subtypes and identifying distinct transcriptional and spatial patterns, we revealed how HRD‐specific macrophage phenotypes contribute to immune surveillance, inflammation, and immune suppression. Furthermore, survival analysis using The Cancer Genome Atlas (TCGA) data highlighted the prognostic value of macrophage subtype signatures, underscoring their roles as key mediators of patient outcomes in HRD‐defined contexts.

## 2. Materials and Methods

### 2.1. scRNA‐seq Data Collection and Integration

Publicly available scRNA‐seq datasets [10] were collected to analyze macrophage populations across HGSOC samples. The single‐cell RNA sequencing dataset used in this study was obtained from Vazquez‐Garcia et al. (Nature, 2022). In that study, multisite tissue biopsies (*n* = 160) were collected from newly diagnosed, treatment‐naïve patients with HGSOC (*n* = 42) undergoing laparoscopy or primary debulking surgery over a 24‐month period. The multisite sampling strategy allowed comprehensive characterization of the spatial and genomic heterogeneity of the tumor immune microenvironment.

HRD subtype annotations (FBI, HRD‐Del, and HRD‐Dup) were obtained from the patient‐level genomic classifications reported by Vazquez‐Garcia et al. [10] based on whole‐genome sequencing. Each single‐cell transcriptomic profile was mapped to its corresponding tumor sample, allowing HRD subtype labels to be assigned to individual cells.

Raw data, including UMI counts and annotations, were obtained directly from original publications. Quality control, annotation, and dataset integration were performed according to established protocols to ensure robust and accurate results. Macrophage cells were identified according to the cell‐type annotations provided in the original dataset metadata. After quality control filtering, a total of 166,895 macrophage cells were retained for downstream analyses, including dimensionality reduction, clustering, and subtype characterization. Subclustering analysis was performed iteratively to identify transcriptionally distinct macrophage subpopulations.

The data were normalized using the Seurat pipeline. The UMI counts for each cell type were log‐transformed and scaled to a factor of 10,000. When UMI counts were unavailable, data were converted to counts‐per‐million (CPM) or transcripts‐per‐million (TPM) for comparability. Highly variable genes (HVGs) were identified using the FindVariable features function. Datasets across multiple batches were integrated using reciprocal PCA (rPCA) and the harmony algorithm, aligning shared features while reducing batch‐specific technical variability.

To further address batch effects, we used scVI (v0.6.8), a probabilistic deep learning model specifically designed for scRNA‐seq analysis [11]. Batch identifiers were included as covariates during model training to correct for technical variability while preserving the biological signals. The corrected latent space is used for downstream dimensionality reduction and clustering analyses.

### 2.2. Dimensionality Reduction, Clustering, and Subclustering

A two‐step process is employed for dimensionality reduction and clustering. The scVI latent space was first projected onto a lower dimensional space (128 dimensions) to capture cell phenotypic variations. UMAP (Rapids.ai cuml v0.12.0) was used to visualize the data in two dimensions [12].

Unsupervised clustering was performed on the scVI latent space using the Leiden algorithm (Cugraph v0.17, resolution = 0.6) to identify major macrophage subtypes.

### 2.3. Functional and Differential Gene Expression Analyses (DEAs)

DEA was conducted to compare macrophage populations across HRD subtypes (FBI, HRD‐Del, HRD‐Dup) and functional states. DEA was performed using the scanpy.tl.rank_genes_groups function (Wilcoxon method), with genes exhibiting a log fold change of > 1.2 and an adjusted *p*‐value of < 0.05 considered significant.

Gene set enrichment analysis (GSEA) [13] was conducted using GSEApy, with pathway annotations sourced from KEGG, GO, Hallmark, and MSigDB databases. Significant enrichment was determined based on a normalized enrichment score (NES) > 1 and adjusted *p*‐value < 0.05. Pathway activation scores for key immune signaling pathways (e.g., JAK‐STAT, TGF‐β) were calculated using the scanpy.tl.score_genes function.

### 2.4. External Validation Using an Independent HGSOC Single‐Cell Dataset

To assess the robustness of the macrophage phenotypes identified in our primary analysis, we performed an external validation using an independent HGSOC single‐cell RNA‐seq dataset reported by Zhang et al. (Sci Adv, 2022). Raw count matrices were downloaded and processed using the same analytical pipeline applied in the discovery dataset. Briefly, cells were filtered using adaptive quality control thresholds based on gene counts, UMI counts, and mitochondrial gene percentages. After normalization and dimensionality reduction, cell‐type annotation was performed based on canonical markers. Macrophages were extracted and reanalyzed using unsupervised clustering to identify transcriptionally distinct macrophage states. Marker genes were identified using the Wilcoxon rank‐sum test, and module‐score analysis was used to evaluate macrophage transcriptional programs corresponding to the six reference macrophage states.

### 2.5. Spatial Transcriptomics Analysis

To explore the spatial organization of macrophage subtypes within the tumor microenvironment, spatial transcriptomic data from publicly available ovarian cancer datasets hosted by the 10x Genomics platform (https://www.10xgenomics.com/datasets) were analyzed. These datasets provide spatially resolved gene expression profiles that enable inference of cell‐type localization within tissue sections.

Spatial data were processed and visualized using Seurat (v4.3) and STUtility following standard workflows. After normalization and spot‐level quality control, macrophage subtype signatures derived from the single‐cell RNA‐seq analysis were projected onto spatial transcriptomic spots using marker gene–based module scoring. This approach allowed us to infer the spatial enrichment patterns of distinct macrophage subtypes across tumor regions.

### 2.6. Survival Analysis

To evaluate the prognostic significance of macrophage subtypes, survival analysis was conducted using the TCGA ovarian cancer cohort. The mean expression of the gene signatures derived from distinct macrophage subtypes was calculated for each patient.

Kaplan–Meier survival curves were generated, and statistical significance was assessed using the log‐rank test. The optimal cutoff values for stratifying high‐ and low‐risk groups were determined using the surv_cutpoint function (survminer package) based on maximally selected rank statistics. Univariate and multivariate Cox proportional hazards (CoxPH) models were used to determine the association between macrophage subtype signatures and disease‐specific survival (DSS) or overall survival (OS) [14, 15]. Covariates such as age and clinical stage were included in multivariate models. Hazard ratios (HRs) and 95% confidence intervals (CIs) were calculated.

### 2.7. Statistical Analysis

All statistical analyses were performed using R Version 4.3.1 and Python Version 3.10.9. Statistical significance was defined as *p*‐value < 0.05. Multiple testing corrections were applied using the false discovery rate (FDR) method for analyses involving more than 20 comparisons [16]. The data distribution was assumed to be normal unless otherwise specified.

Key Python packages included Scanpy, NumPy, Pandas, and SciPy for scRNA‐seq analysis, whereas survival analysis was conducted using the “survival” and “survminer” R packages. Visualizations were generated using ggplot2, matplotlib, and Seaborn. Detailed descriptions of the statistical tests and experimental procedures are provided in the corresponding figure legends.

## 3. Results

### 3.1. HRD‐Driven Reprogramming of Macrophage Function and Immune Microenvironment

To elucidate the spatial heterogeneity and expression dynamics of key genes across macrophage populations, we obtained a publicly available single‐cell RNA sequencing dataset of ovarian cancer. After extracting macrophage cells and performing rigorous quality control and integration, we obtained a total of 166,895 high‐quality macrophages [10]. Dimensionality reduction and clustering analysis were performed to examine the distribution of cells from different tissue origins, ethnic backgrounds, age groups, and HRD subtypes (Figures 1(a), 1(b), 1(c), and 1(d)). The results revealed minimal distribution differences among macrophages from various tissues, ethnicities, or age groups. In contrast, macrophages exhibited distinct distribution preferences across samples with different HRD subtypes, which could be categorized into the FBI, HRD‐Del, and HRD‐Dup groups. HRD subtype classification was defined at the tumor‐sample level based on mutational signatures inferred from whole‐genome sequencing. Furthermore, the cell density plot confirmed noticeable differences in macrophage distribution among HRD subtypes, revealing distinct immune surveillance and repair mechanisms in each HRD group (Figure 1(e)). The gene expression correlation heatmap elucidated the relationships between differentially expressed genes across the subtypes, revealing distinct coexpression modules that contributed to the functional specialization observed within these populations (Figure 1(f)). These distinct density patterns suggest that HRD‐driven alterations can shape macrophage function and contribute to immune microenvironment reprogramming.

FIGURE 1Spatial and transcriptional heterogeneity of macrophages across HRD subtypes in high‐grade serous ovarian cancer (HGSOC). (a–c) UMAP projections displaying macrophage populations across tissue types (a), ethnic groups (b), and patient age categories (c), highlighting the diversity within macrophage populations. (d) UMAP visualization categorizing macrophages by homologous recombination deficiency (HRD) subtypes: functional BRCA1/2 inactivation (FBI), HRD‐Del (deletions), and HRD‐Dup (duplications). Distinct clustering patterns indicate subtype‐specific transcriptional and spatial characteristics. (e) Density plots for macrophage populations across HRD subtypes (FBI, HRD‐Del, HRD‐Dup), illustrating the enriched spatial zones and highlighting the unique immune surveillance and functional dynamics of each HRD subtype. (f) Gene expression correlation heatmap depicting coexpression modules among key genes, revealing HRD subtype‐specific gene clusters that contribute to functional macrophage specialization. (g) Pathway enrichment analysis across macrophage populations, showing differential activation of immune signaling modules (e.g., JAK‐STAT, TGF‐β, and MAPK pathways) for each HRD subtype. (h) Dot plot summarizing the mean expression levels and fraction of macrophage cells involved in major pathway modules, stratified by FBI, HRD‐Del, and HRD‐Dup subtypes.

**Figure:**
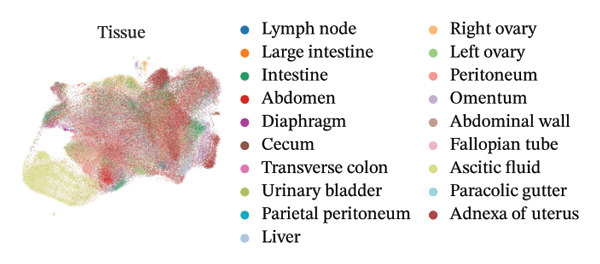
(a)

**Figure:**
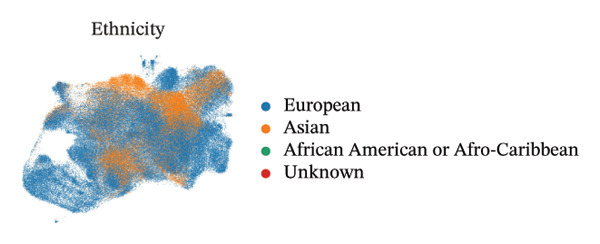
(b)

**Figure:**
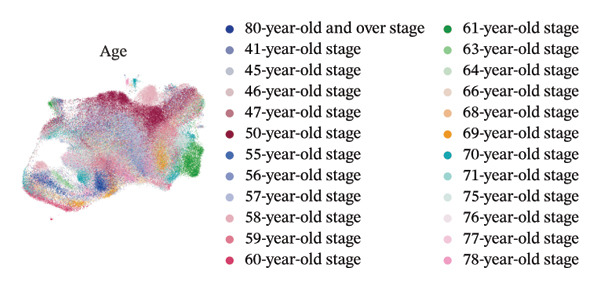
(c)

**Figure:**
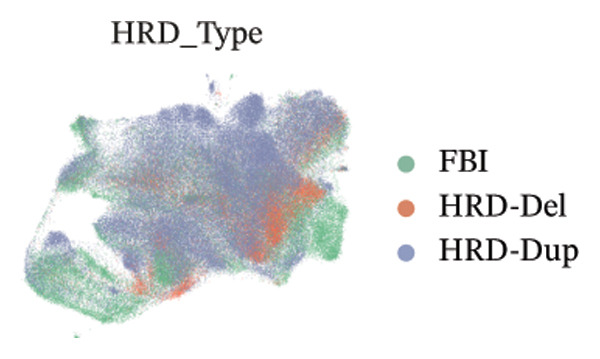
(d)

**Figure:**
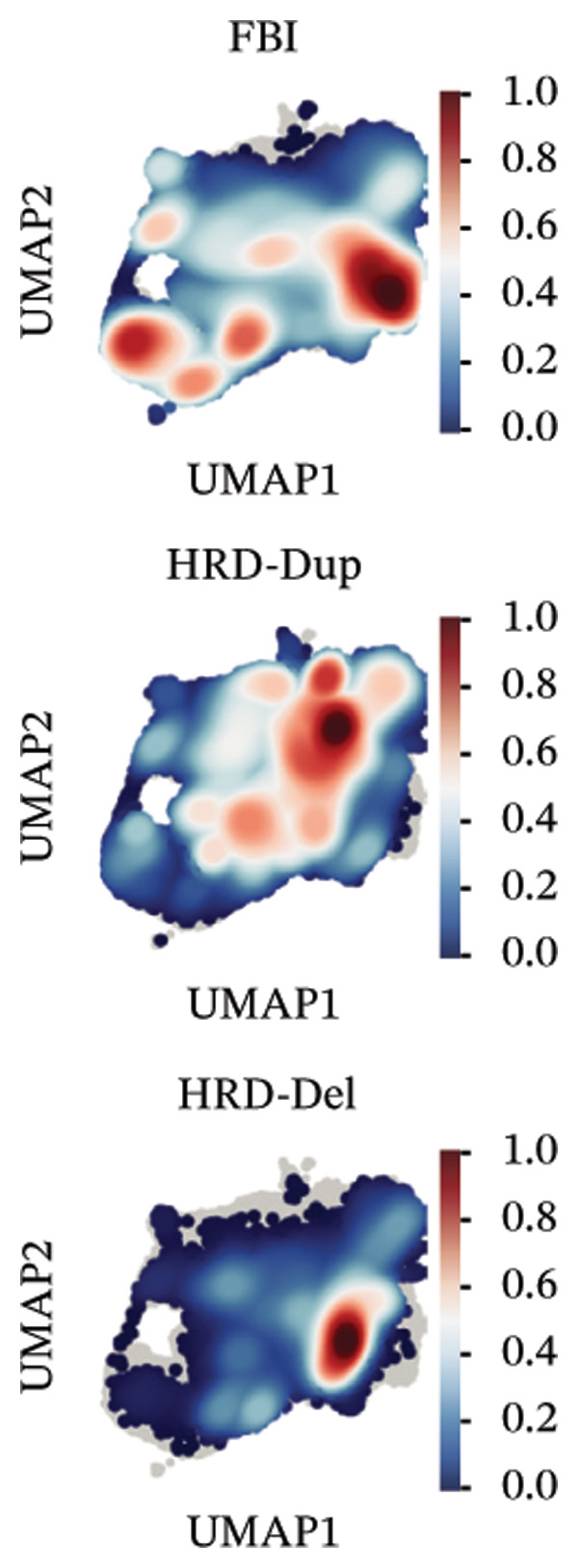
(e)

**Figure:**
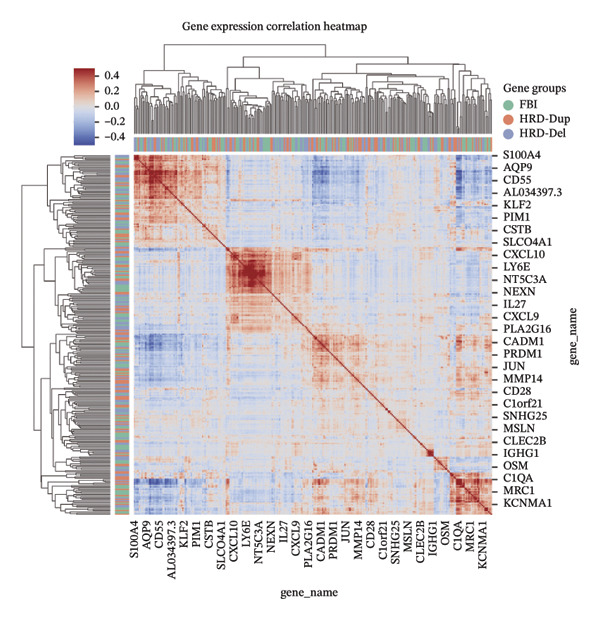
(f)

**Figure:**
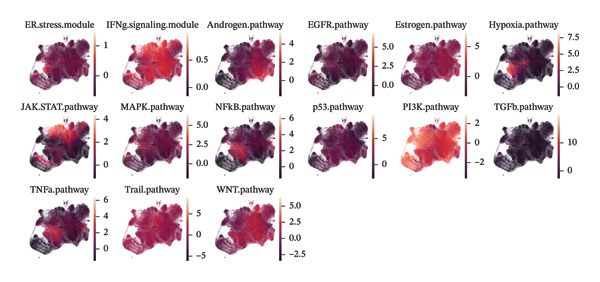
(g)

**Figure:**
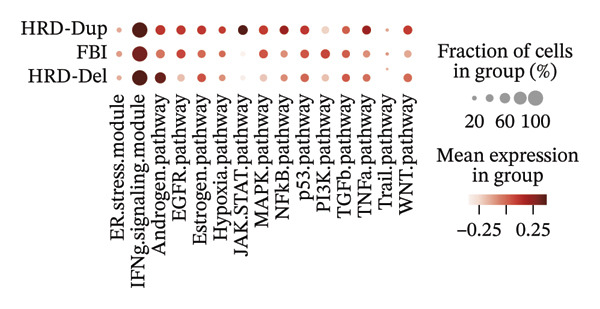
(h)

Significant differences in gene expression were observed among different HRD subtypes. In the FBI macrophage group, RNASE1, CTSB, CD163, S100A8, and CD36 were upregulated. These genes exhibited regionally clustered expression patterns, indicating their role in cellular degradation, immune regulation, and inflammatory responses, particularly in macrophage‐like myeloid populations. RNASE1 and CTSB displayed diffuse, yet enriched expression across multiple loci, suggesting their involvement in cellular degradation processes, whereas S100A8 and CD36 were linked to inflammatory cascades and lipid metabolism (Supporting Figure 1A). In HRD‐Del macrophages, the upregulated genes included HLA‐DRB5, GSN, HLA‐DQA1, HLA‐DPB1, and HLA‐DQB1, which were integral to antigen presentation and immune signaling. UMAP visualization highlighted a substantial overlap in expression, which correlated with antigen‐presenting cell clusters, suggesting that a coordinated immune response potentially enhances adaptive immunity. HLA‐DQA1 and HLA‐DPB1 formed particularly intense expression zones, emphasizing their critical role in T‐cell activation and antigen processing, whereas GSN appeared to be more dispersed, indicating its supportive function in cytoskeletal reorganization (Supporting Figure 1B). In HRD‐Dup macrophages, interferon‐stimulated genes (ISGs), such as IF16, ISG15, MX1, LY6E, and IFI44L, were significantly upregulated. UMAP projections indicated robust antiviral responses in these macrophages, marked by elevated expression in specific niches. ISG15 and MX1 were particularly prominent, suggesting strong Type I interferon pathway activation, which is consistent with enhanced innate immune defense mechanisms. The coordinated expression of ISGs in HRD‐Dup macrophages may reflect their specialized roles in innate immune activation and antiviral defense (Supporting Figure 1C).

Pathway enrichment analysis revealed several key signaling modules, including JAK‐STAT, MAPK, TGF‐β, and TNF‐α pathways, each exhibiting differential activation across the HRD subtypes. The JAK‐STAT and MAPK pathways showed increased activity in HRD‐Del macrophages, suggesting enhanced cytokine signaling and stress response mechanisms in these populations. Conversely, the TGF‐β pathway was particularly pronounced in HRD‐Dup macrophages, implicating this pathway in immune suppression and extracellular matrix remodeling, potentially contributing to an immune evasion phenotype (Figure 1(g)).

Collectively, ovarian cancer patients with different HRD subtypes exhibit significant differences in macrophage distribution preferences, gene expression profiles, and signaling pathway enrichment. These findings suggest that HRD subtypes drive functional and transcriptional reprogramming of macrophages. Therefore, it is essential to further annotate these macrophage subpopulations and their gene expression features, and to explore the impact of distinct macrophage subsets on the immune microenvironment and prognosis of patients.

### 3.2. Macrophage Clustering Revealed Functionally Distinct Subtypes in the Tumor Microenvironment

To further characterize the macrophage subpopulations across different HRD subtypes, we performed unsupervised clustering and identified six distinct macrophage subsets. Each subset was annotated based on its uniquely highly expressed marker genes. UMAP projections identified subpopulations enriched in C1Q, FCN1, MARCO, MKI67, MMP9, and S100A9 marker genes (Figure 2(a)). These distinct clusters highlight macrophage heterogeneity and suggest functional specialization in processes, such as immune activation, tissue remodeling, and proliferation.

FIGURE 2Macrophage subtype characterization and functional clustering in high‐grade serous ovarian cancer. (a) UMAP visualization of six distinct macrophage subtypes identified through single‐cell RNA sequencing analysis. (b) Gene expression correlation matrix showing coexpression relationships between key marker genes across identified macrophage subtypes. Red indicates positive correlation, and blue indicates negative correlation. (c) Differential expression analysis of marker genes for each macrophage subtype, showing distinct top‐scoring genes when compared to all other subtypes. (d) UMAP visualization depicting macrophage subtypes color‐coded by cluster identity, emphasizing the spatial heterogeneity and functional diversity within the tumor microenvironment. (e) Heatmap summarizing mean z‐scores for key marker gene expression across macrophage subtypes, providing insights into the transcriptional programs governing their functional roles. (f) Density plots depicting the spatial distribution and relative abundance of each macrophage subtype within the tumor microenvironment, revealing distinct localization patterns.

**Figure:**
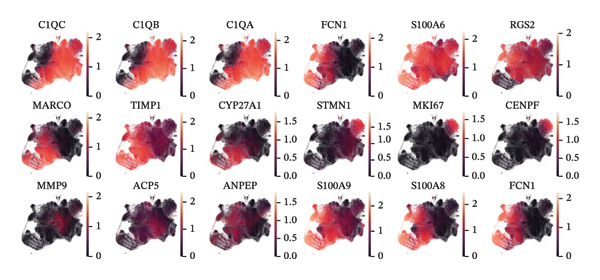
(a)

**Figure:**
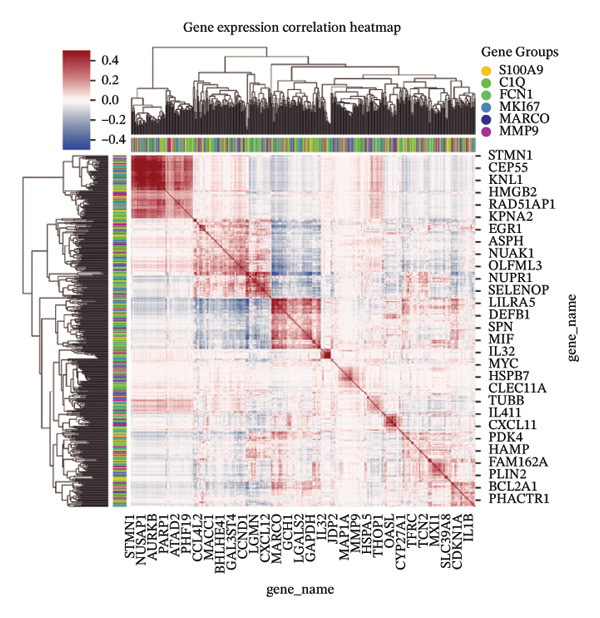
(b)

**Figure:**
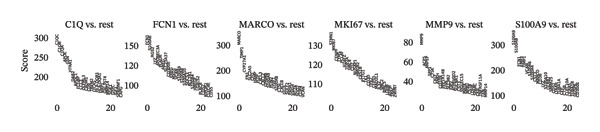
(c)

**Figure:**
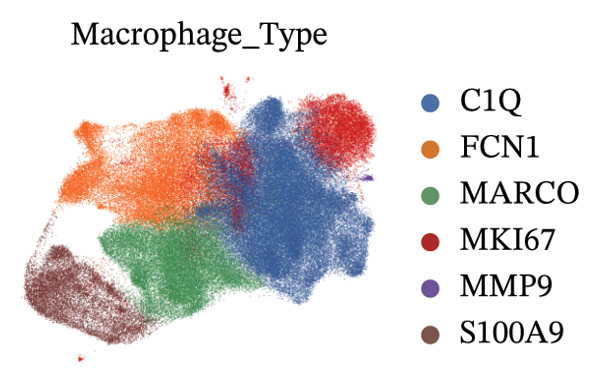
(d)

**Figure:**
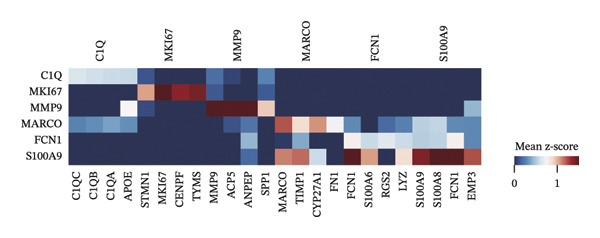
(e)

**Figure:**
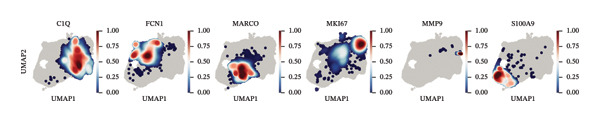
(f)

Gene expression correlation analysis revealed coexpression modules across macrophage clusters, providing insights into the relationships between the differentially expressed genes. Genes associated with immune activation (C1Q and S100A9), matrix remodeling (MMP9 and MARCO), and proliferation (MKI67) formed tight clusters, suggesting that coordinated transcriptional programs drive macrophage functional specialization (Figure 2(b)). Differential expression analysis further delineated the top marker genes for each cluster. The C1Q cluster exhibited high expression of C1QA, C1QB, and C1QC, which is indicative of classical complement activation. The FCN1 cluster is enriched in FCN1 and other immune response genes, highlighting its role in innate immunity. The MARCO cluster showed increased expression of MARCO and related genes involved in phagocytosis and extracellular matrix interactions. Proliferative macrophages (MKI67 cluster) display elevated MKI67 and CENPF expression, reflecting their active cell cycle state. In the MMP9 cluster, MMP9 and TIMP1 were upregulated, consistent with their roles in matrix degradation and tumor invasion. Finally, the S100A9 cluster was characterized by S100A8 and S100A9, genes linked to inflammation and stress responses (Figures 2(c) and 2(d)). Each of these macrophage clusters exhibited unique gene expression patterns, reflecting their distinct roles in immune response, matrix remodeling, proliferation, and antigen presentation (Figure 2(e)). Density plots for each macrophage subtype provided further insight into their distribution (Figure 2(f)).

To evaluate the reproducibility of macrophage phenotypes identified in our discovery cohort, we analyzed an independent HGSOC single‐cell RNA‐seq dataset reported by Zhang et al. (2022). After quality control filtering, 51,553 cells were retained for downstream analysis (Supporting Figure 2A). Among these, 6916 macrophages were extracted based on canonical macrophage markers and subjected to unsupervised clustering (Supporting Figure 2B). Reanalysis of the macrophage compartment revealed six transcriptionally distinct macrophage states that corresponded closely to those identified in our primary dataset, including C1Q‐like, FCN1‐like, MARCO‐like, MKI67‐like, MMP9‐like, and S100A9‐like macrophages (Supporting Figures 2C and 2D). Each state displayed marker gene expression patterns consistent with our original findings. For example, C1QA/C1QB/C1QC were enriched in C1Q‐like macrophages, FCN1 and HLA Class II genes characterized FCN1‐like macrophages, MARCO/APOE/CD163 marked MARCO‐like macrophages, MKI67/CENPF/TOP2A defined proliferative macrophages, MMP9/TIMP1 highlighted the matrix‐remodeling subset, and S100A8/S100A9 identified inflammatory macrophages (Supporting Figure 2E). Module‐score analysis further confirmed that these transcriptional programs were preferentially enriched in their corresponding macrophage states, supporting the reproducibility of these macrophage phenotypes across independent datasets (Supporting Figure 2F).

### 3.3. Macrophage Proportions and Functional Associations Across HRD Subtypes

Group distribution analysis revealed significant variations in the proportions of macrophage subtypes across samples, highlighting the distinct immune microenvironment compositions among all samples (Figure 3(a), Supporting Table 1). Pie chart visualization further delineates these differences. In the FBI group, C1Q‐positive macrophages dominated the population (38.0%), suggesting their role in complement activation and immune surveillance. In contrast, the HRD‐Del group displayed a higher proportion of FCN1 macrophages (41.2%), indicating their involvement in innate immune processes and antigen presentation. The HRD‐Dup group was enriched in MARCO‐positive macrophages (20.6%), consistent with their enhanced roles in tissue remodeling and tumor‐associated macrophage (TAM) activity. Although the S100A9 and MMP9 subtypes were present in smaller proportions, they were particularly prominent in HRD‐Del, suggesting a specialized role in inflammation and extracellular matrix degradation (Figure 3(b)).

FIGURE 3Distribution and functional analysis of macrophage subtypes across HRD categories in high‐grade serous ovarian cancer. (a) Group distribution plot showing the relative proportions of macrophage subtypes across individual samples, demonstrating heterogeneity in immune microenvironment composition. (b) Pie charts representing macrophage subtype proportions for FBI, HRD‐Del, and HRD‐Dup tumors. C1Q macrophages dominate in FBI (38.0%), FCN1 macrophages are enriched in HRD‐Del (41.2%), and MARCO macrophages are abundant in HRD‐Dup (20.6%). (c) Quantitative comparison of macrophage subtype proportions across HRD categories. (d) Stacked bar plot showing ethnicity‐specific distributions of macrophage subtypes, revealing variations in immune cell composition across different ethnic backgrounds. (e) Dot plot displaying pathway associations of macrophage subtypes across HRD subtypes. Key pathway enrichments include JAK‐STAT in MKI67 macrophages (HRD‐Del), complement activation in C1Q macrophages (FBI), and TGF‐β signaling in MARCO macrophages (HRD‐Dup). Dot size represents the fraction of cells, and color indicates mean expression.

**Figure:**
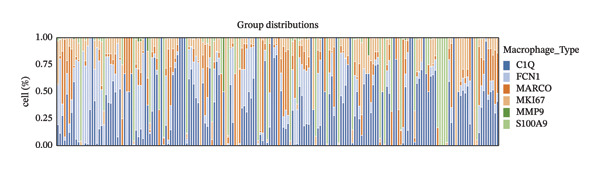
(a)

**Figure:**
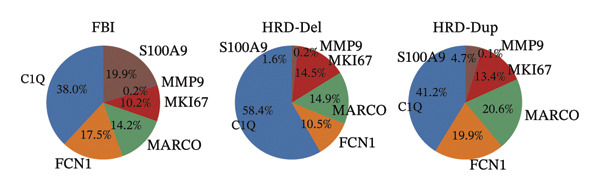
(b)

**Figure:**
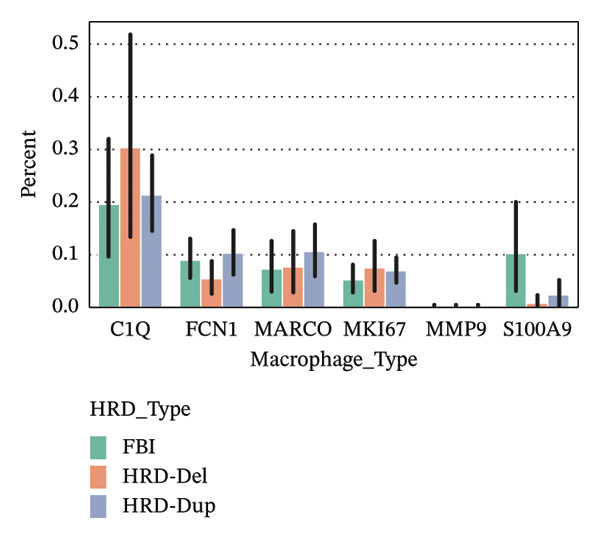
(c)

**Figure:**
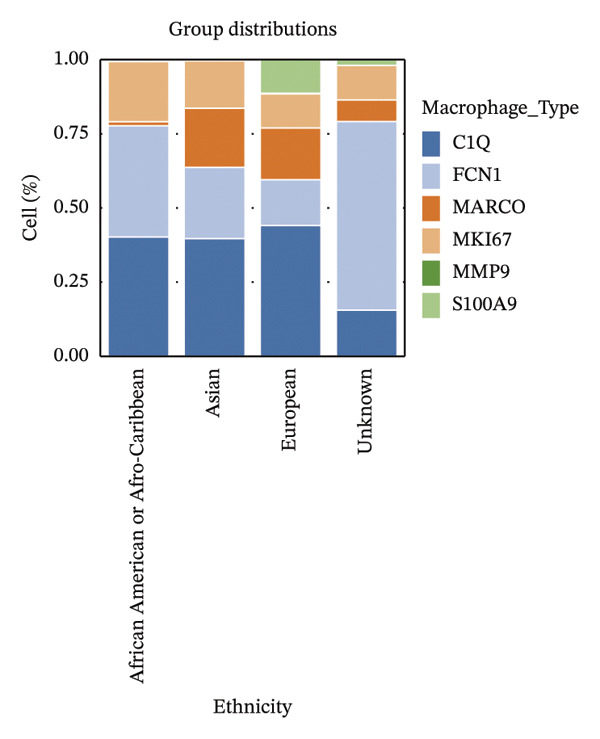
(d)

**Figure:**
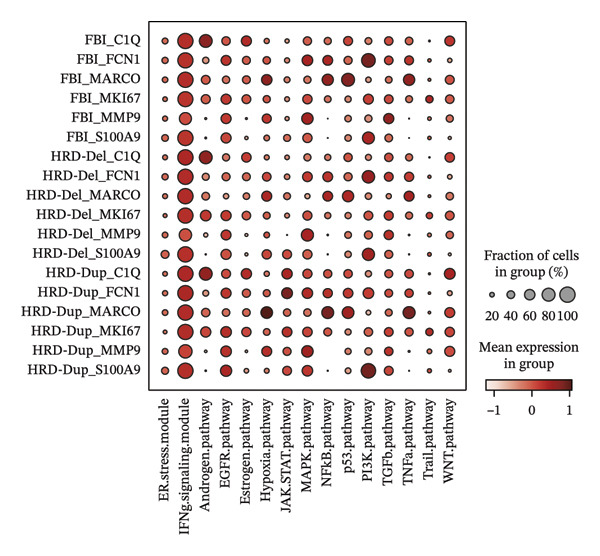
(e)

A bar plot comparison quantitatively assessed the proportions of macrophage subtypes across the HRD subtypes. C1Q macrophages were significantly enriched in FBI, whereas MKI67 macrophages, which have proliferative activity, showed elevated expression of HRD‐Del. MARCO macrophages were particularly abundant in HRD‐Dup, further emphasizing tissue remodeling and immunosuppressive niche formation within this subgroup (Figure 3(c)).

Ethnicity‐specific distributions revealed variations in the macrophage proportions across diverse ethnic groups. African American/Caribbean and Asian populations showed higher enrichment of C1Q macrophages, whereas European samples exhibited increased proportions of FCN1 and MARCO subtypes. This observation underscores the interplay between the genetic background and macrophage functional adaptation in response to HRD‐driven alterations (Figure 3(d)).

Pathway enrichment analysis provided insights into the functional correlations among macrophage subtypes across HRD categories. Notably, C1Q macrophages in the FBI were enriched in IFN signaling and complement activation pathways, supporting their role in immune surveillance. MKI67 macrophages in HRD‐Del were associated with the JAK‐STAT and stress–response pathways, highlighting their proliferative and inflammatory nature. In HRD‐Dup, MARCO macrophages exhibited strong associations with the TGF‐β and WNT pathways, indicative of immune suppression and extracellular matrix remodeling (Figure 3(e)).

Collectively, these findings demonstrate that HRD‐driven genomic subtypes shape macrophage composition and functional pathways in a subtype‐specific manner, contributing to the heterogeneity of the tumor immune microenvironment. These insights provide a foundation for understanding how macrophage phenotypes influence tumor progression and potential therapeutic responses.

### 3.4. Spatial Distribution and Correlation of Macrophage Subtypes Across HRD Subtypes

The correlation heatmap revealed distinct relationships between macrophage subtypes across the HRD categories. C1Q‐positive macrophages inversely correlated with MKI67 and MMP9 populations, suggesting mutually exclusive roles in immune surveillance and tissue remodeling. In contrast, MARCO and S100A9 subtypes showed positive correlations in HRD‐Dup samples, reflecting their coenrichment in the immunosuppressive and inflammatory niches (Figure 4(a)). These patterns indicate that macrophage subtype interactions are influenced by HRD‐driven genomic alterations that shape the immune microenvironment.

FIGURE 4Spatial distribution and subtype correlations of macrophages across HRD subtypes in high‐grade serous ovarian cancer (HGSOC). (a) Correlation heatmap showing relationships between macrophage subtypes across HRD categories. (b) Immunohistochemistry (IHC) image of a tumor section showing spatial organization of macrophage subtypes within the tumor microenvironment, highlighting localized macrophage populations in immune and stromal compartments. (c) Density plots for macrophage spatial distributions across HRD subtypes. (d) Subtype‐specific density plots of macrophages. C1Q macrophages localize in central immune niches, MKI67 macrophages display a broad distribution reflecting their proliferative nature, MARCO macrophages are enriched in stromal regions associated with tissue remodeling, and S100A9 macrophages form localized clusters, indicative of inflammatory activity.

**Figure:**
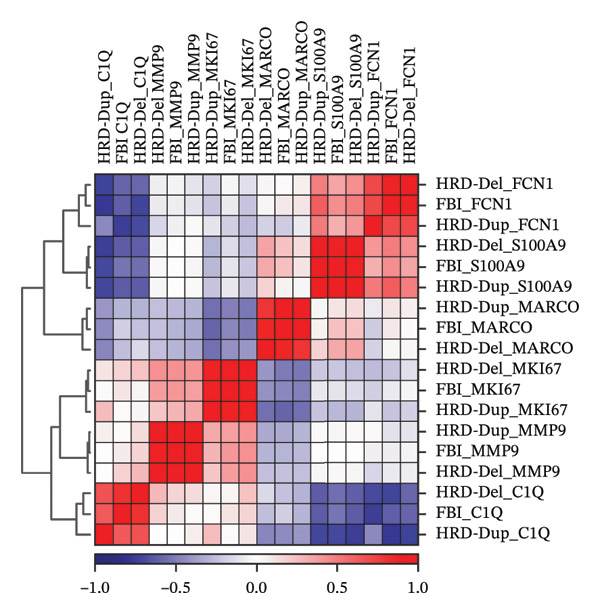
(a)

**Figure:**
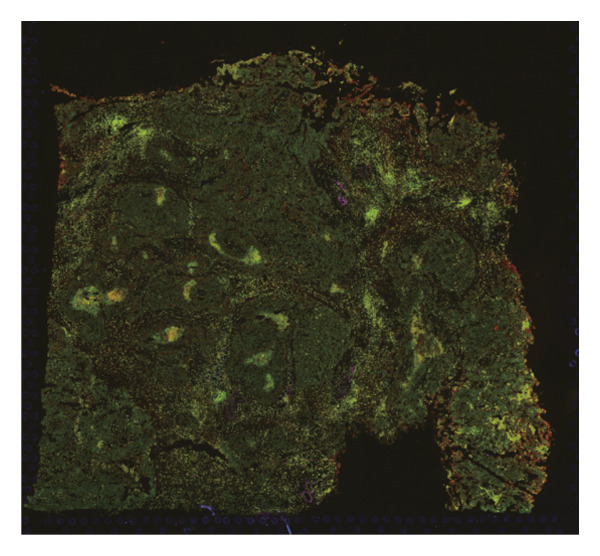
(b)

**Figure:**
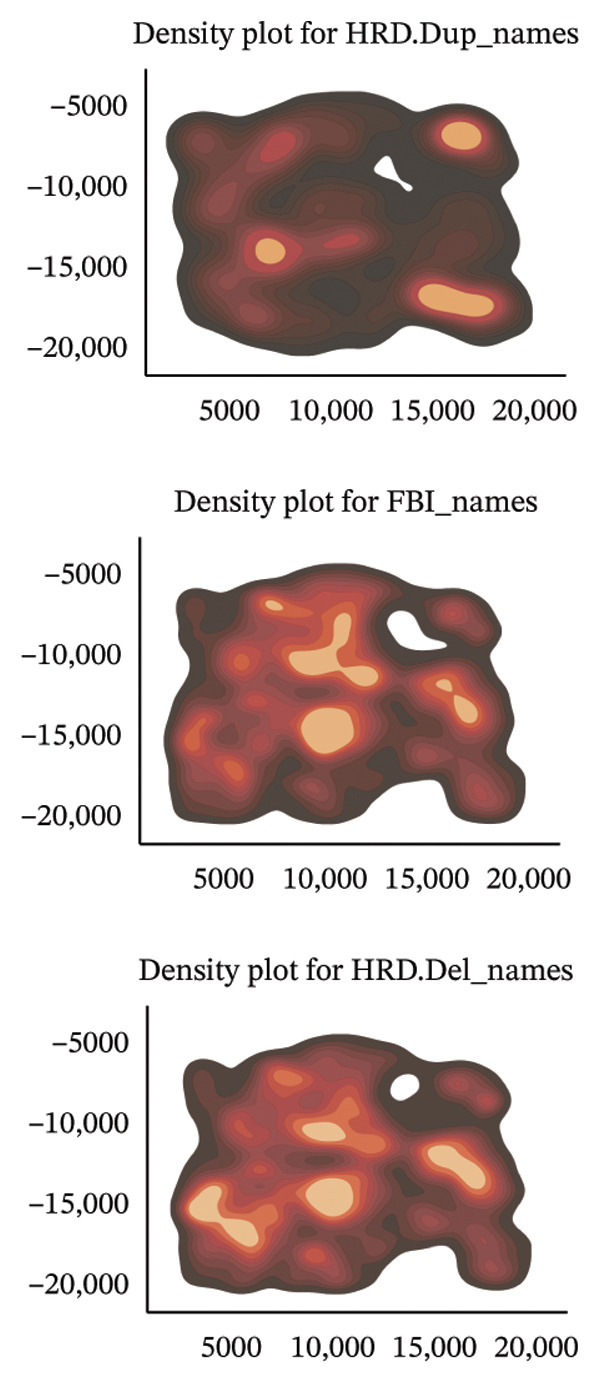
(c)

**Figure:**
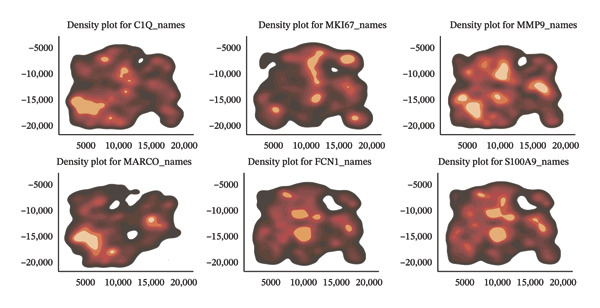
(d)

To understand the spatial architecture of macrophage populations in the tumor microenvironment and their association with HRD‐driven genomic subtypes, we analyzed public spatial data and macrophage subtype correlations. Spatial transcriptomic datasets were used to infer macrophage spatial distribution patterns, and immunohistochemistry images were used for visual illustration (Figure 4(b)). This spatial compartmentalization suggests functional specialization of macrophages within the microenvironment, which is likely driven by genomic instability and tissue‐specific cues. Density plots demonstrated HRD‐specific macrophage distribution. In HRD‐Dup tumors, macrophage subtypes displayed broader spatial infiltration across the stromal and tumor compartments, indicative of extensive tissue remodeling and immune suppression. In contrast, FBI and HRD‐Del tumors showed macrophage clustering in the focal regions, suggesting localized immune activation and functional compartmentalization of macrophages (Figure 4(c)).

The subtype‐specific density maps further clarified these spatial patterns. C1Q macrophages, which are associated with complement activation, were densely localized in the central tumor niches, whereas MKI67‐positive macrophages exhibited a widespread distribution, consistent with their proliferative activity. MARCO macrophages are enriched in stromal regions, reflecting their roles in extracellular matrix remodeling and immunosuppression. MMP9 and S100A9 macrophages formed discrete clusters in areas likely to undergo inflammation and tissue remodeling, while FCN1 macrophages were more diffusely distributed, highlighting their roles in antigen presentation and immune surveillance (Figure 4(d)).

Together, these findings demonstrate that the spatial distribution of macrophage subtype and inter‐subtype correlations was influenced by HRD‐driven genomic instability. The observed compartmentalization highlights the functional diversity of macrophages in shaping the tumor microenvironment and suggests that HRD subtypes dictate the immune landscape organization, which may have implications for spatially targeted therapies.

### 3.5. Survival Analysis Highlighted the Prognostic Impact of HRD‐Specific Macrophage Subtypes

To evaluate the clinical relevance of HRD‐specific macrophage subtypes, survival analysis was conducted using TCGA data. DSS and OS were analyzed based on gene signatures derived from single‐cell macrophage subtypes and their distribution across HRD subtypes [17, 18].

Distinct survival patterns were observed in the HRD‐specific macrophage subtype analysis. Patients with HRD‐Dup macrophage subtype signatures exhibited significantly improved survival outcomes, with HRs of 0.63 (95% CI: 0.48–0.82, *p* < 0.001) for DSS and 0.66 (95% CI: 0.51–0.84, *p* = 0.001) for OS. These results suggest a protective role for macrophages in the HRD‐Dup group. In contrast, HRD‐Del and FBI macrophage subtypes did not demonstrate significant associations with survival, indicating variability in their contribution to prognosis across HRD‐driven macrophage states (Figures 5(a) and 5(b)).

FIGURE 5Survival analysis of HRD macrophage subtypes and their prognostic significance in HGSOC patients. (a) Kaplan–Meier curves showing disease‐specific survival (DSS) stratified by HRD macrophage subtypes. HRD‐Dup macrophage signatures demonstrate significantly improved survival (HR: 0.63, 95% CI: 0.48–0.82, *p* < 0.001). (b) Overall survival (OS) analysis across HRD macrophage subtypes, with HRD‐Dup showing favorable outcomes (HR: 0.66, 95% CI: 0.51–0.84, *p* = 0.001). (c) DSS analysis based on single‐cell‐derived macrophage subtype signatures. High C1Q expression correlates with worse outcomes (HR: 1.28, 95% CI: 0.97–1.68, *p* = 0.056), while elevated MKI67 expression shows improved survival (HR: 0.73, 95% CI: 0.56–0.94, *p* = 0.016). (d) OS analysis for macrophage subtype signatures, highlighting the contrasting effects of C1Q (HR: 1.28, 95% CI: 1.01–1.63, *p* = 0.045) and MKI67 (HR: 0.74, 95% CI: 0.58–0.94, *p* = 0.017) expression on patient outcomes.

**Figure:**
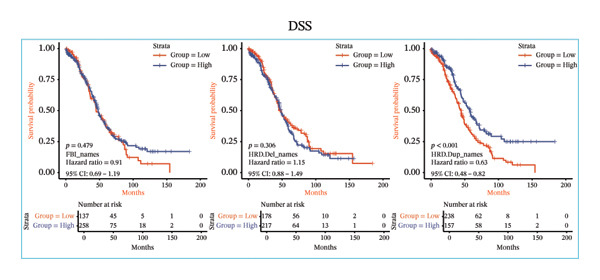
(a)

**Figure:**
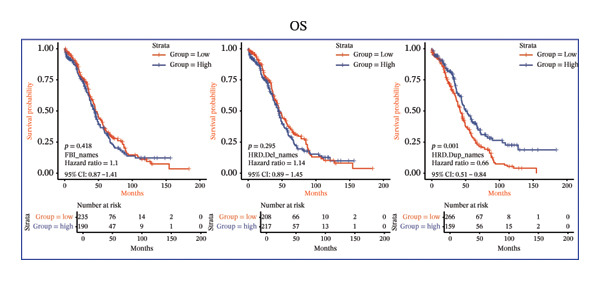
(b)

**Figure:**
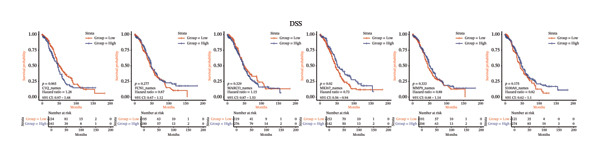
(c)

**Figure:**
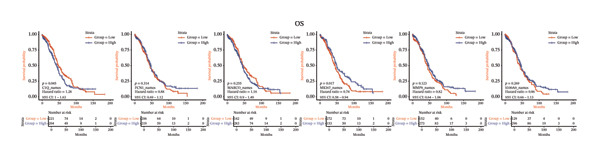
(d)

Survival analysis based on single‐cell‐derived gene signatures of macrophage subtypes revealed subtype‐specific survival trends. C1Q‐enriched macrophage signatures were associated with poor survival outcomes. Patients with high C1Q expression had HRs of 1.28 (95% CI: 0.97–1.68, *p* = 0.056) for DSS and 1.28 (95% CI: 1.01–1.63, *p* = 0.045) for OS, suggesting that these macrophages may contribute to immune suppression and complement activation, negatively affecting survival (Figures 5(c) and 5(d)).

In contrast, MKI67‐enriched macrophage signatures were significantly associated with improved outcomes. Patients with high MKI67 expression displayed better survival, with HRs of 0.73 (95% CI: 0.56–0.94, *p* = 0.016) for DSS and 0.74 (95% CI: 0.58–0.94, *p* = 0.017) for OS. This suggests that proliferative macrophage subtypes play an active role in immune activation and tumor control, contributing to favorable patient outcomes (Figures 5(c) and 5(d)).

Other HRD‐specific macrophage subtype signatures, including MARCO, FCN1, MMP9, and S100A9, were not consistently associated with survival. However, MARCO trended to have a modest impact on DSS, suggesting a context‐dependent role in tissue remodeling and immune suppression (Figures 5(c) and 5(d)).

Collectively, these results underscore the prognostic significance of HRD‐specific macrophage subtypes and their functional heterogeneity in HGSOC. The protective role of macrophages in HRD‐Dup tumors highlights the potential for immune activation within specific HRD contexts. Conversely, the negative association between C1Q macrophage signatures and survival outcomes suggests their role in immunosuppression. These insights emphasize the importance of HRD‐specific macrophage subtypes in shaping patient prognosis and provide a rationale for the development of macrophage‐targeted therapeutic strategies tailored to the HRD‐driven immune microenvironment.

## 4. Discussion

Our study not only highlights how HRD influences macrophage function and spatial architecture in HGSOC but also emphasizes the clinical implications of these findings. By leveraging single‐cell transcriptomics, spatial data, and survival analysis, we delineated how the HRD subtypes—namely, FBI, HRD‐Del (deletions), and HRD‐Dup (duplications)—reprogram macrophage phenotypes, spatial organization, and functional roles in the tumor microenvironment. Importantly, our findings resonate with and expand upon existing literature, providing novel insights into HRD‐driven immune modulation. Recent single‐cell studies have provided important insights into the cellular composition and immune heterogeneity of HGSOC. For example, Deng et al. [18] constructed a single‐cell atlas of metastatic HGSOC, revealing extensive immune and stromal heterogeneity within the tumor microenvironment. Similarly, integrated immunogenomic analyses by Gronauer et al. [17] further highlighted the complexity of immune cell states and their potential implications for immunotherapy response. These studies collectively emphasize the importance of dissecting immune cell diversity at single‐cell resolution in ovarian cancer.

This study demonstrated subtype‐specific macrophage transcriptional profiles that aligned with the findings of recent immuno‐oncology studies. For instance, FBI macrophages are enriched in lipid metabolism and inflammatory regulatory genes (e.g., CD36 and S100A8). These findings parallel recent observations in the metabolic reprogramming of TAMs, as described by Ren et al., where lipid metabolism has been implicated in immune suppression and tumor progression [19]. The upregulation of antigen‐presentation genes (e.g., HLA‐DQA1 and HLA‐DPB1) in HRD‐Del macrophages underscores their adaptive immune response roles, consistent with insights from Wang et al., who linked enhanced antigen presentation to favorable tumor immunogenicity in HRD‐driven cancers [20]. Importantly, the reproducibility of these macrophage states was further supported by an independent HGSOC single‐cell RNA‐seq dataset, indicating that the macrophage transcriptional programs identified in this study represent robust immune phenotypes rather than dataset‐specific clustering artifacts.

The spatial distribution of the macrophage subtypes revealed compartmentalized functions that reflected the underlying genomic and microenvironmental cues. For example, C1Q macrophages’ localization in central tumor niches in FBI tumors mirrors the findings of Senent et al., who reported complement‐mediated immune suppression within immune niches [21]. The widespread spatial infiltration of MKI67 macrophages in HRD‐Del tumors aligns with studies by Mantovani et al., which highlighted the role of proliferative TAMs in tumor control and immune activation [22]. These spatial patterns reinforce our findings and underscore the functional specialization of macrophages based on the tumor genomic context.

The association between HRD and increased M2‐like macrophage infiltration suggests a potential therapeutic strategy for targeting macrophage polarization. Modulating the tumor microenvironment to reprogram TAMs from a protumoral (M2‐like) to an antitumoral (M1‐like) phenotype could enhance treatment efficacy. Strategies such as inhibition of the CSF‐1/CSF‐1R pathway have been proposed to reduce M2 macrophage populations and are currently under investigation. Unlike the original study by Vazquez‐Garcia et al., which focused primarily on mutational processes and T‐cell dynamics, our macrophage‐centered reanalysis highlights HRD‐associated transcriptional and spatial heterogeneity of TAMs.

Although our study provides valuable insights, it is limited by the availability of comprehensive datasets linking HRD status with macrophage phenotypes. Future research should focus on integrating multiomics data to validate these findings and explore the mechanistic pathways underlying macrophage polarization in HRD tumors. In addition, clinical trials assessing therapies targeting macrophage polarization in patients with HRD‐positive HGSOC are warranted. In conclusion, our study highlights the significant role of HRD in shaping the immune landscape of HGSOC, particularly concerning macrophage polarization and spatial distribution. These findings open new avenues for the development of macrophage‐targeted therapies tailored to the genomic context of ovarian cancer, potentially improving patient outcomes.

## Author Contributions

Hua Lan: data acquisition, data curation, formal analysis, and writing–review and editing. Fang Xu: data acquisition and writing–review and editing. Linshuang Li: software, validation, and writing–review and editing. Xin Wei: writing–review and editing. Minghua Li: funding acquisition, project administration, conceptualization, methodology, and writing–review and editing.

## Funding

This work was supported by the Natural Science Foundation of Hunan Province (Grant Nos. 2023JJ40073, 2026JJ82241, 2026JJ82018).

## Disclosure

The work reported in the article had been performed by the authors. All authors edited and approved the final version of the manuscript.

## Ethics Statement

The authors have nothing to report.

## Conflicts of Interest

The authors declare no conflicts of interest.

## Supporting Information

Additional supporting information can be found online in the Supporting Information section.

## Supporting information


**Supporting Information 1** Supporting Figure 1: UMAP projections illustrating upregulated gene expression across various HRD macrophage subtypes: HRD (A), FBI (B), HRD‐Del (C), and HRD‐Dup.

**Supporting Information 2** Supporting Figure 2. External validation of macrophage transcriptional states in an independent HGSOC single‐cell RNA‐seq dataset. (A) UMAP visualization of major cell populations identified in the independent HGSOC single‐cell RNA‐seq dataset reported by Zhang et al. (2022), including malignant cells and multiple stromal and immune cell types. (B) UMAP projection of macrophages extracted from the dataset, revealing six transcriptionally distinct macrophage states: C1Q‐like, FCN1‐like, MARCO‐like, MKI67‐like, MMP9‐like, and S100A9‐like macrophages. (C) Dot plot showing the expression patterns of representative marker genes across the six macrophage states. Dot size indicates the percentage of cells expressing the gene, and color intensity represents average expression levels. (D) Heatmap displaying the top marker genes for each macrophage state, highlighting distinct transcriptional programs across macrophage subtypes. (E) Feature plots illustrating the spatial expression patterns of representative marker genes (C1QA, FCN1, MARCO, MKI67, MMP9, and S100A9) across macrophage populations in the UMAP embedding. (F) Module‐score analysis of macrophage subtype‐specific gene signatures, demonstrating preferential enrichment of each transcriptional program in the corresponding macrophage state.

**Supporting Information 3** Supporting Table 1. Relative abundance of macrophage subtypes in the discovery cohort.

## Data Availability

The data used in this study were obtained from the public databases, which have been stated in the part of the material and methods.
