# Supplementary figures and images for: HRD‐Driven Reprogramming of Macrophage Function and Spatial Architecture in High‐Grade Serous Ovarian Cancer
Source: Genet Res (Camb). 2026 May 9;2026:7364793. doi: 10.1155/genr/7364793 (PMC13157315; doi:10.1155/genr/7364793)

**A**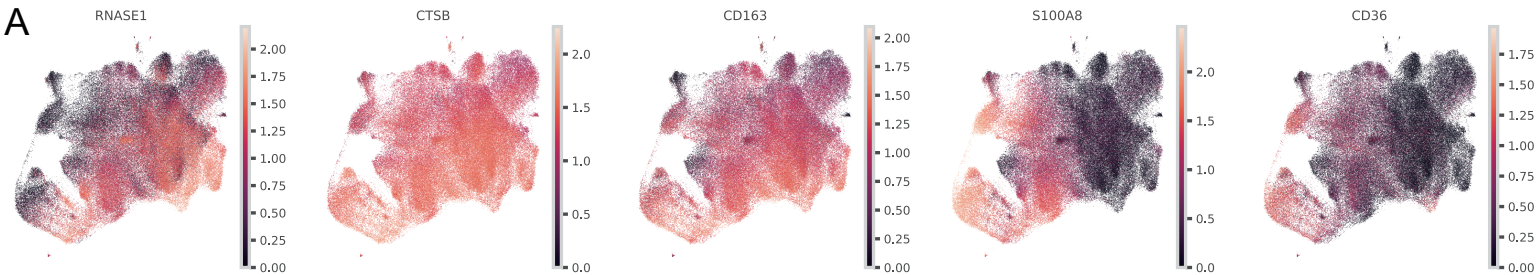**B**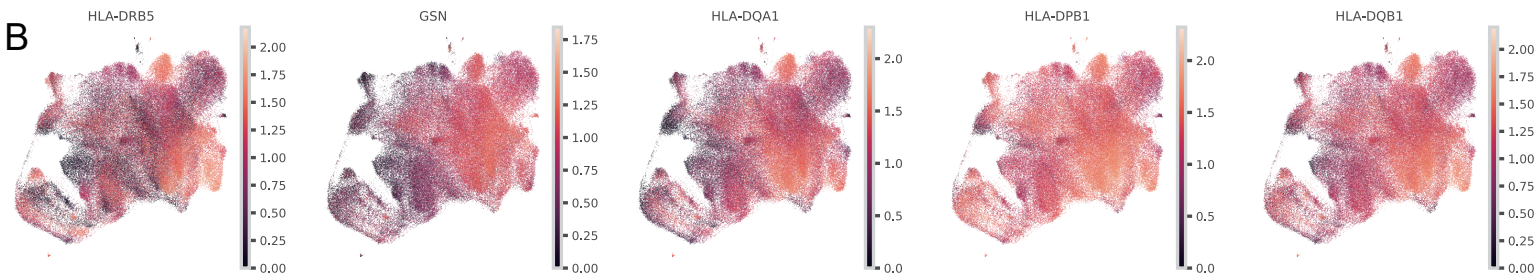**C**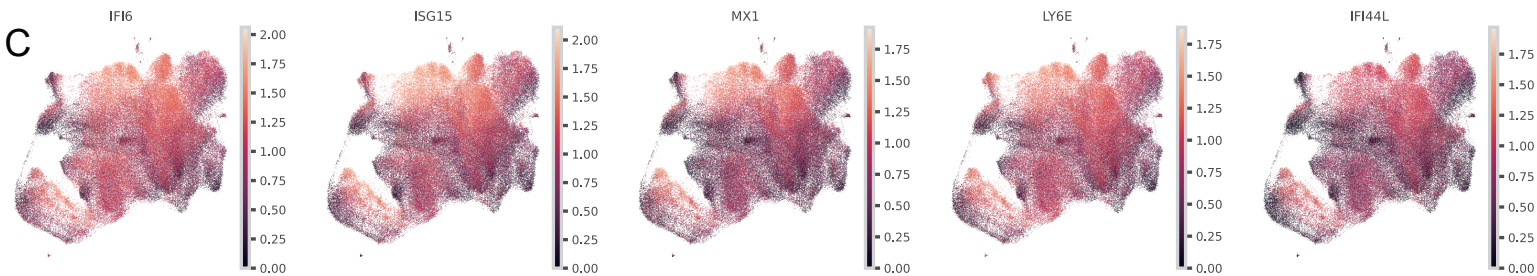

Supplement: Supplementary file 1 — Supporting Information 1 Supporting Figure 1: UMAP projections illustrating upregulated gene expression across various HRD macrophage subtypes: HRD (A), FBI (B), HRD‐Del (C), and HRD‐Dup. [file GENR-2026-7364793-s001.pdf]

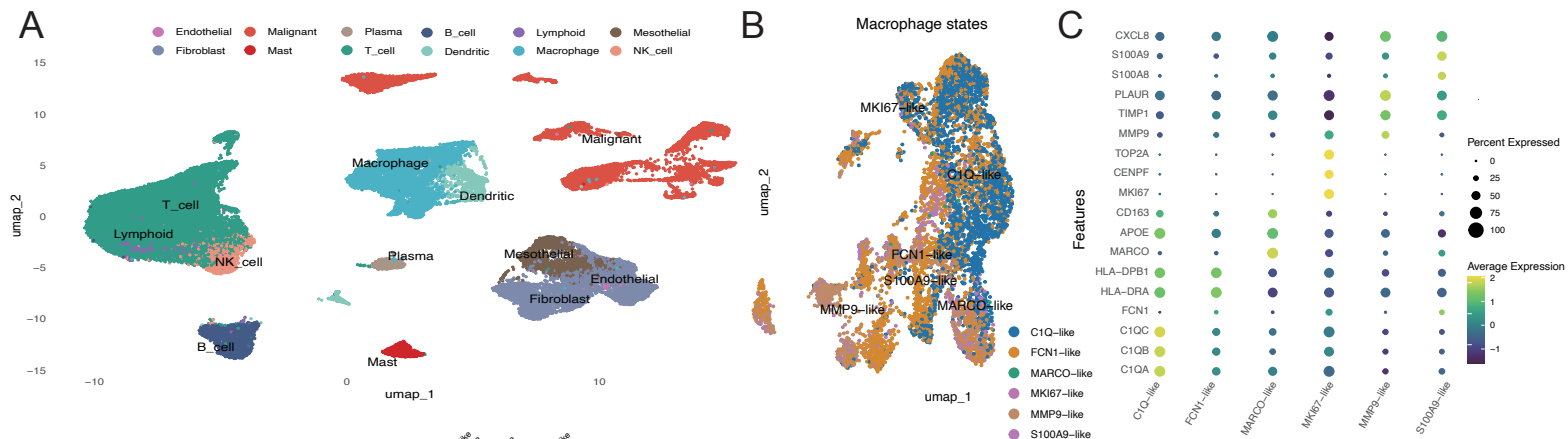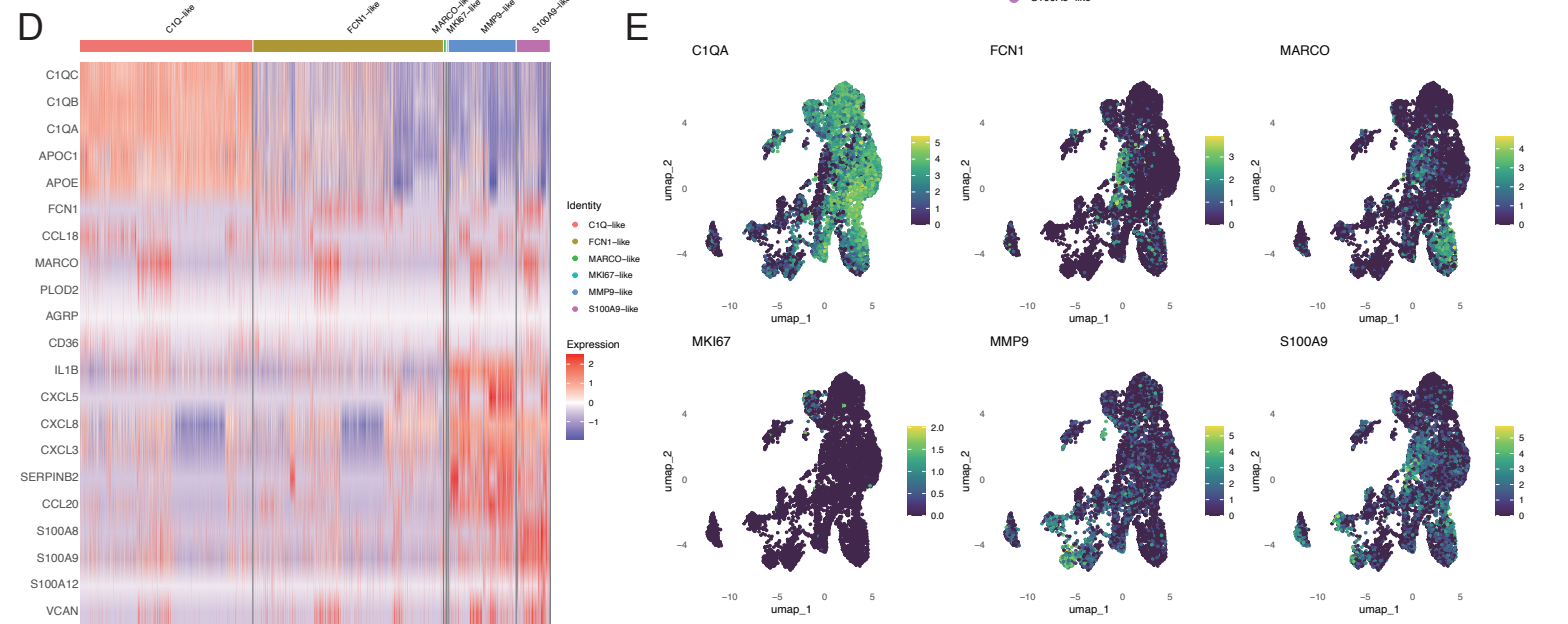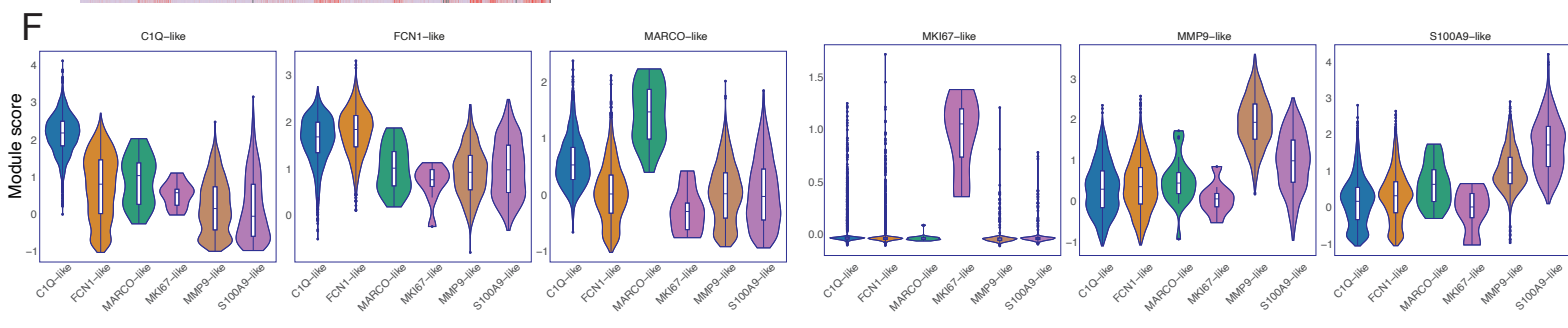

Supplement: Supplementary file 2 — Supporting Information 2 Supporting Figure 2. External validation of macrophage transcriptional states in an independent HGSOC single‐cell RNA‐seq dataset. (A) UMAP visualization of major cell populations identified in the independent HGSOC single‐cell RNA‐seq dataset reported by Zhang et al. (2022), including malignant cells and multiple stromal and immune cell types. (B) UMAP projection of macrophages extracted from the dataset, revealing six transcriptionally distinct macrophage states: C1Q‐like, FCN1‐like, MARCO‐like, MKI67‐like, MMP9‐like, and S100A9‐like macrophages. (C) Dot plot showing the expression patterns of representative marker genes across the six macrophage states. Dot size indicates the percentage of cells expressing the gene, and color intensity represents average expression levels. (D) Heatmap displaying the top marker genes for each macrophage state, highlighting distinct transcriptional programs across macrophage subtypes. (E) Feature plots illustrating the spatial expression patterns of representative marker genes (C1QA, FCN1, MARCO, MKI67, MMP9, and S100A9) across macrophage populations in the UMAP embedding. (F) Module‐score analysis of macrophage subtype‐specific gene signatures, demonstrating preferential enrichment of each transcriptional program in the corresponding macrophage state. [file GENR-2026-7364793-s002.pdf]
