# Supplementary material for: HRD‐Driven Reprogramming of Macrophage Function and Spatial Architecture in High‐Grade Serous Ovarian Cancer
Source: Genet Res (Camb). 2026 May 9;2026:7364793. doi: 10.1155/genr/7364793 (PMC13157315; doi:10.1155/genr/7364793)
Supplement: Supplementary file 3 — Supporting Information 3 Supporting Table 1. Relative abundance of macrophage subtypes in the discovery cohort. [file GENR-2026-7364793-s003.rtf]

Supplementary Table 1. Relative abundance of macrophage subtypes in the discovery cohort.
Macrophage subtype	 Cell number	 Proportion (%)	
C1Q-like	68,194	50.9	
FCN1-like	30,747	23.0	
MARCO-like	30,040	22.4	
MKI67-like	20,820	15.6	
S100A9-like	13,801	10.3	
MMP9-like	264	0.2	
